# Load Capacity and Displacement of Recycled Concrete and Self-Insulation Block Masonry Wall

**DOI:** 10.3390/ma13040863

**Published:** 2020-02-14

**Authors:** Huizhi Zhang, Jifeng Liu, Yang Yue, Xiuqin Cui, Yuezong Lian

**Affiliations:** 1 College of Architecture and Civil Engineering, Sanming University, Sanming 365004, China; ph_dliujifeng@126.com (J.L.); yybeijing@126.com (Y.Y.); cuixiuqin@163.com (X.C.); lianyuezong@163.com (Y.L.); 2 Key Laboratory of Engineering Materials & Structure Reinforcement (Sanming University), Fujian Province University, Sanming 365004, China

**Keywords:** Load capacity, displacement, recycled concrete and self-insulation block, masonry wall

## Abstract

In order to discuss the load capacity and displacement of masonry constructed with recycled concrete and self-insulation blocks, one type of 10.6 MPa compressive strength block and three kinds of mortar with M15, M10, and M5 compressive strengths are selected. The constitutive model and corresponding parameters selection of different materials in the ABAQUS numerical simulation are analyzed, and the numerical simulation analysis and experimental tests of the load capacity and displacement of masonry constructed with mortars of different strengths are carried out. The results show that masonry compression failure is controlled by the mortar or block that has the lower compressive strength. The displacement of masonry increases with the mortar compressive strength increase, and the higher mortar compressive strength is beneficial for improving the load capacity and displacement of masonry. Reasonable selection of the constitutive model and parameters will help to obtain reasonable results for the ABAQUS numerical simulation. Construction quality and loading method will affect the load capacity and displacement of the masonry. The above conclusion can provide reference for the engineering application of recycled concrete and self-insulation blocks.

## 1. Introduction

In recent years, with the rapid development of China’s economy, the improvement of urban and rural living environments has led to a large amount of building demolition and reconstruction. On the one hand, more than 1 billion tons of construction and demolition waste (CDW) are generated every year. About 40% of this CDW is structural concrete, which could generate about 400 million tons of recycled aggregates (RA) [1,2]. On the other hand, new building construction needs a large amount of coarse and fine concrete aggregate. The traditional sources of coarse and fine concrete aggregate are quarrying in mountains and excavating in river beds, both of which cause great environmental damage. At the same time, the demand for metal products is increasing with the development of the economy. Some metal mining enterprises have increased their productivity, but pay less attention to environmental protection. In the process of metal mining, the separation operation is beneficiation, where the useful target component content is low and cannot be used for production, i.e., tailings, which are largely produced and reserved and take up a lot of land and induce risks such as dam break, acid solution filtration, and environmental pollution. The metal tailings could be activated by grinding or physical and chemical treatment methods and used as cementing material for concrete. This is one of the most important ways of using tailings as a resource, and has good economic, social, and environmental benefits [3,4].

Economic development means that people have a higher requirement in terms of the comfort of their living environment. However, according to statistics, the energy consumption of China’s construction industry accounts for about 30% of the total energy consumption of society, and this proportion is increasing with the annual growth of the national construction area. However, more than 95% of the buildings in China are high energy consumption buildings. The increasing energy consumption demand of the construction industry has led to a huge energy crisis in China. Therefore, building energy conservation has become an important part of social energy conservation and has huge potential for energy saving. The method of self-insulation of exterior walls has gained interest recently because of its outstanding advantages, such as the service life of the building, low cost, convenient construction, safety, and reliability. Reducing the heat transfer coefficient of the self-insulation systems of exterior walls is the key to improving the level of building energy conservation. Therefore, it is of great significance in terms of economic and environmental protection value to find a type of energy-saving building wall material that can comprehensively utilize recycled concrete and metal tailings as building materials and improve the technical level of wall energy-saving [5].

Some scholars have studied the compressive properties of new block masonry. Longo, et.al. experimentally determined the mechanical and the thermal capacities of FRCM-matrices by compressive tests, flexural tests and thermal conductivity measurements [6]. Xiao et al. have studied the compressive strength of natural concrete and recycled concrete hollow-block masonry and analyzed the effect of fly ash and polypropylene fiber on the compressive strength of recycled concrete hollow-block masonry [7]. Xia et al. have studied the effect of mortar strength on the compressive properties of light block masonry and analyzed the damage characteristics of masonry [8,9]. Gan et al. have tested the compressive properties of large cavity concrete porous brick masonry [10]. Lin et al. have tested and analyzed the compressive and shear properties of five types of new material masonry and compared them to the compressive and shear properties of common brick masonry [11]. Leurenco and Pina-Henriques have analyzed the compressive strength of masonry by analytic and continuous numerical methods [12]. Chaimoon and Attard have proposed a numerical shear failure model of unreinforced masonry [13]. Binda et al. have carried out compression-shear tests of the masonry constructed by two types of stone and two types of construction method and put forward the corresponding numerical analysis model [14]. The research of references [12,13,14] shows that it is feasible to study the mechanical properties of recycled self-insulation concrete block masonry by numerical simulation. The parameter selection, boundary conditions, grid division, constitutive model, etc., used in these articles can provide useful references for this study. However, there are limited reports on the load capacity and displacement of recycled concrete and self-insulation block masonry; therefore, the load capacity and displacement of the new self-insulation block masonry should be further studied in order to provide reference for engineering design.

References [12,13,14] show that numerical simulation is one of the most effective ways to study engineering problems. On the basis of analyzing the constitutive model and parameter selection of mortar and recycled concrete and self-insulation blocks, ABAQUS numerical software (6.13 Version, Dassault Systèmes Simulia Corp., Providence, RI, USA.) is used to simulate the influence law of masonry compressive strength and the displacement of masonry in order to provide a reference for the engineering application of the type of new recycled concrete and self-insulation block presented by the authors.

## 2. Numerical Simulation Method and Parameters Selection

**Figure 1:**
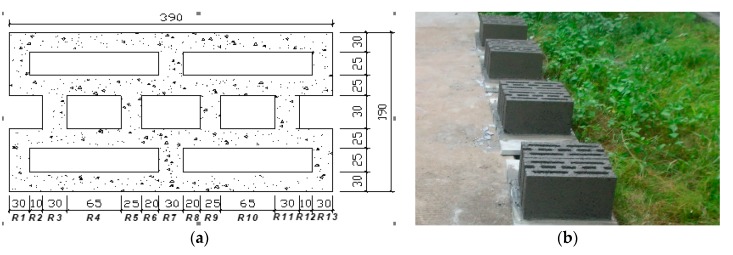
Details of the recycled concrete and self-insulation blocks (**a**) and the real blocks (**b**).

The recycled concrete and self-insulation block size is the same as that shown in the literature [1,2,3]. The size of the block is length × width × height = 390 mm × 190 mm × 190 mm. Three rows of holes are designed; the thickness of the holes is 25 mm, the thickness of the outer hole wall is 35 mm or 30 mm, the outer row to the vertical rib wall thickness is 30 mm, and the other rib wall thicknesses are each 25 mm. Per cubic meter, the recycled concrete mix of this type of block is 1205 kg of recycled coarse aggregate (i.e., 100% replacement rate for natural coarse aggregate), 408 kg of 42.5 grade cement, 72 kg of activated zinc and lead tailing powder, which is a 15% replacement rate for cement, 150.5 kg of natural sand, 150.5 kg of recycled fine aggregate (i.e., 50% replacement rate for natural sand), and 120% volume fraction of vitrified microspheres. For details of the recycled concrete and self-insulation blocks and real blocks, see Figure 1. It is calculated from Figure 1a that the void ratio of this kind of multi-hole block is 29.4%, and the thermal conductivity and heat transfer coefficient of this kind of block masonry wall are 35.2% lower and the total thermal resistance is 54.7% higher than that of ordinary concrete block walls under the same conditions. Moreover, the thermal conductivity of the recycled concrete block is much lower than that of the ordinary concrete after mix proportion optimization. At the same time, the excellent thermal insulation effect of three rows of holes leads to the great energy-saving effect of the recycled concrete block.

The experimental compressive strength of block concrete material is 25 MPa, elasticity modulus is 2.55 × 10^4^ MPa, and the Poisson ratio is 0.3. The compressive strength of recycled concrete and self-insulation blocks is 10.6 MPa, shear strength is 1.1 MPa, elasticity modulus is 503 MPa, and the Poisson ratio is 0.28. The parameters of three types of mortar with different strengths, obtained from technical sheets, are as follows: (1) M15 mortar; compressive strength is 15 MPa and elastic modulus is 12 × 10^3^ MPa, (2) M10 mortar; compressive strength is 10 MPa and elastic modulus is 8.5 × 10^3^ MPa., and (3) M5 mortar; compressive strength is 5 MPa and elastic modulus is 6 × 10^3^ MPa. All of the above parameters will be adopted in the ABAQUS numerical simulation.

A plastic damage model is adopted to simulate the properties of recycled concrete and self-insulation blocks and mortar. The plasticity model parameters are as follows: The dilation angle *ψ* = 30°, the eccentricity *ϵ* = 0.1, the ultimate compressive strength ratio of biaxial compression and uniaxial compression σ_b0_/σ_c0_ = 1.16, the invariable stress ratio *K_c_* = 0.667, and the viscosity parameter *μ* = 0.0005 [2].

The strain values of the block concrete and the mortar are obtained from the corresponding stress by the concrete or mortar constitutive, i.e., the adopted plasticity model and the inelastic strain are calculated by Equations (1) and (2).

(1) $${\tilde{\varepsilon }}^{in}=\varepsilon_{c}-\varepsilon_{0c}^{el}$$

(2) $$\varepsilon_{0c}^{el}=\sigma_{c}/E_{0}$$

Here, ${\tilde{\varepsilon }}^{in}$ is inelastic strain, $\varepsilon_{c}$ is compression strain, $\varepsilon_{0c}^{el}$ is initial elastic strain, $\sigma_{c}$ is compression stress, MPa, and $E_{0}$ is Young’s modulus, MPa.

Taking the proportion, $\beta_{c}$, of plastic strain, ${\tilde{\varepsilon }}_{c}^{pl}$, in nonlinear elastic strain, ${\tilde{\varepsilon }}_{c}^{in}$ is 0.4; the compression damage parameter, $d_{c}$, could be calculated by Equation (3).

(3) $$d_{c}=\frac{(1-\beta_{c}){\tilde{\varepsilon }}_{c}^{in}E_{0}}{\sigma_{c}+(1-\beta_{c}){\tilde{\varepsilon }}_{c}^{in}E_{0}}$$

The compression recovery factor takes the default, i.e., $w_{c}=1$. 

Similarly, taking the proportion, $\beta_{t}$, of the plastic strain, ${\tilde{\varepsilon }}_{t}^{pl}$, in nonlinear elastic strain, ${\tilde{\varepsilon }}_{t}^{in}$ is 0.7; the tension damage parameter, $d_{t}$, could be calculated by Equation (4).

(4) $$d_{t}=\frac{(1-\beta_{t}){\tilde{\varepsilon }}_{t}^{in}E_{0}}{\sigma_{c}+(1-\beta_{t}){\tilde{\varepsilon }}_{t}^{in}E_{0}}$$

Tension recovery factor takes the default, i.e., $w_{t}=0$ [15].

**Figure 2:**
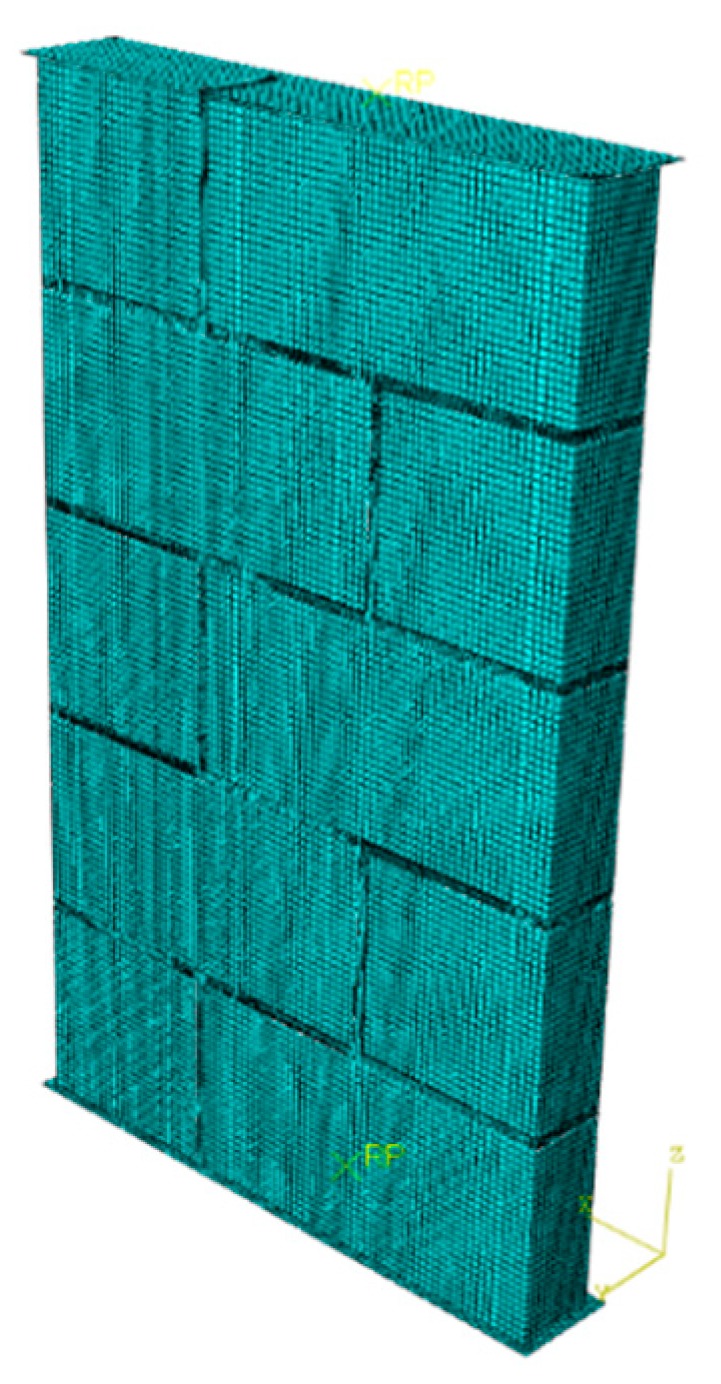
Model and grid partition.

For simulating the actual test conditions, a rigid plate is used as a loading tool to transmit force through the contact surface. The size of the rigid plate exceeds 10 mm for each side of the block. The influence of geometric nonlinearity is considered by statics analysis. The friction coefficient between the rigid plate and the concrete block is 0.1. Loading mode is displacement control. There are 246,966 C3D8R units used in the model, and the unit density is 8 mm for the block and 6 mm for the mortar. The numerical simulation model and grid partition are shown in Figure 2.

**Figure 3:**
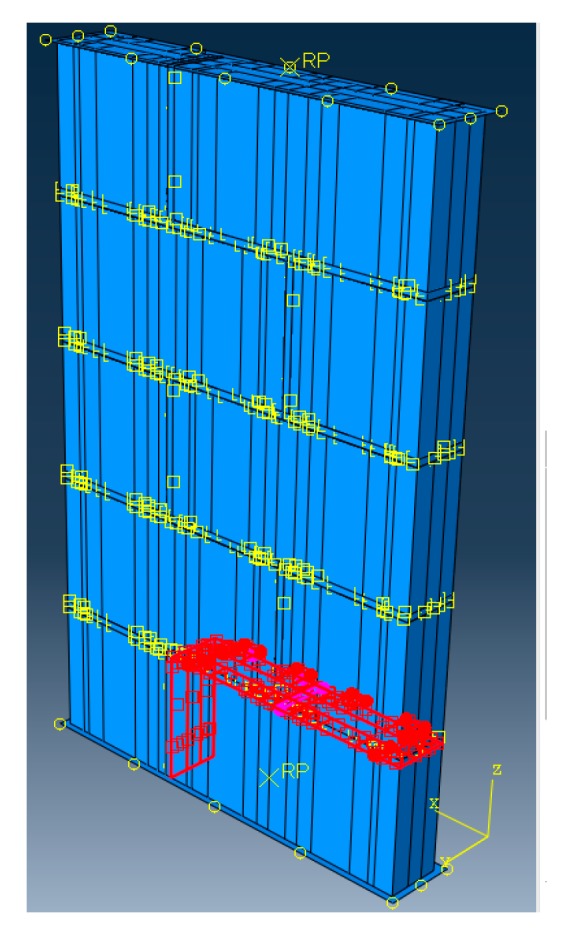
Model constraints and interface processing.

The lower part of the wall is supported by a rigid plate, and the upper part is a rigid plate under pressure. The rigid plate is used to transmit force between the walls. Cohesive contact between mortar and block is taken into account in order to consider the bond relationship between the mortar and the block interface. The contact property mutual parameters between block and mortar are set as follows: The elastic parameters are based on the unit stiffness of the contact surface, and the damage parameters are as follows: the maximum normal stress and shear stress are both 10^7^ N at initiation, the type of evolution is energy and the softening mode is linear, the normal, 1st and 2nd, shear fracture energies are both 63.7 J, and the viscosity coefficient of stabilization is 5 × 10^−5^. Model constraints and interface processing are detailed in Figure 3.

## 3. Analysis of Numerical Simulation Results

**Figure 4:**
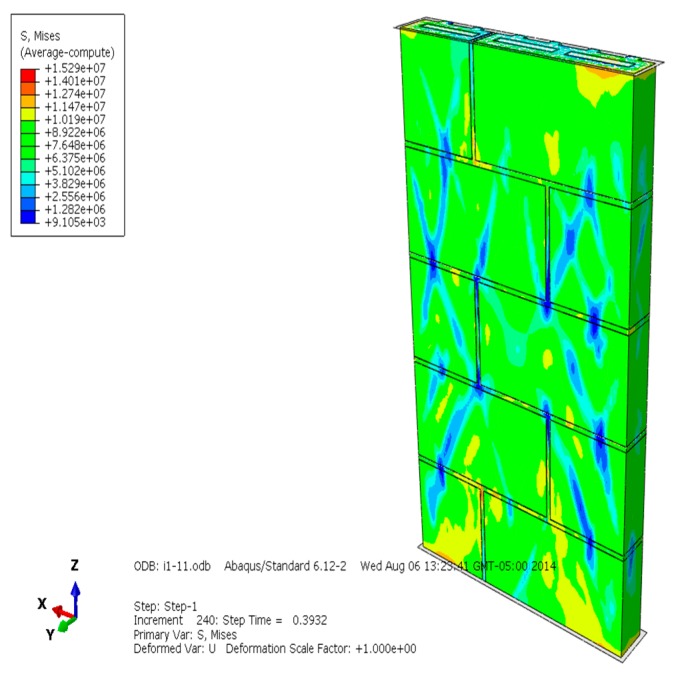
Mises equivalent stress diagram of masonry constructed with M15 mortar.

**Figure 5:**
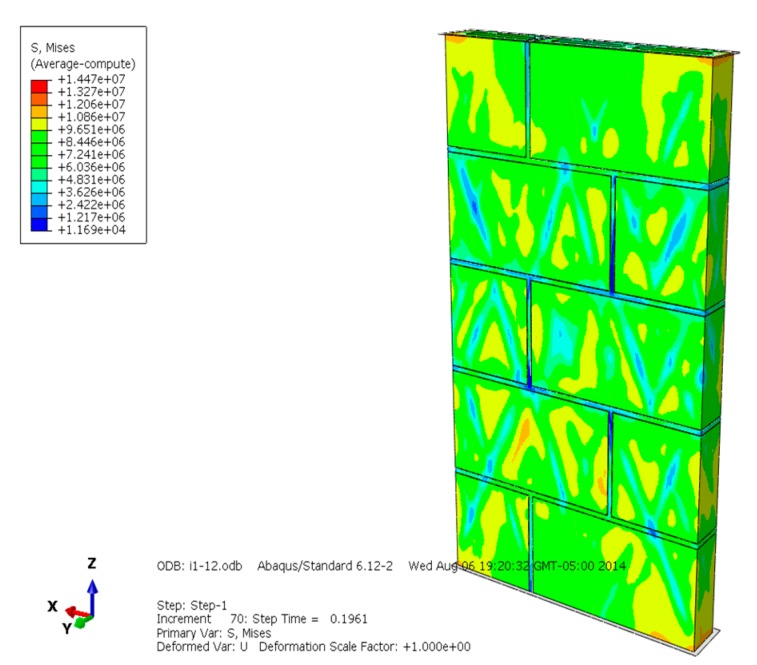
Mises equivalent stress diagram of masonry constructed with M10 mortar.

**Figure 6:**
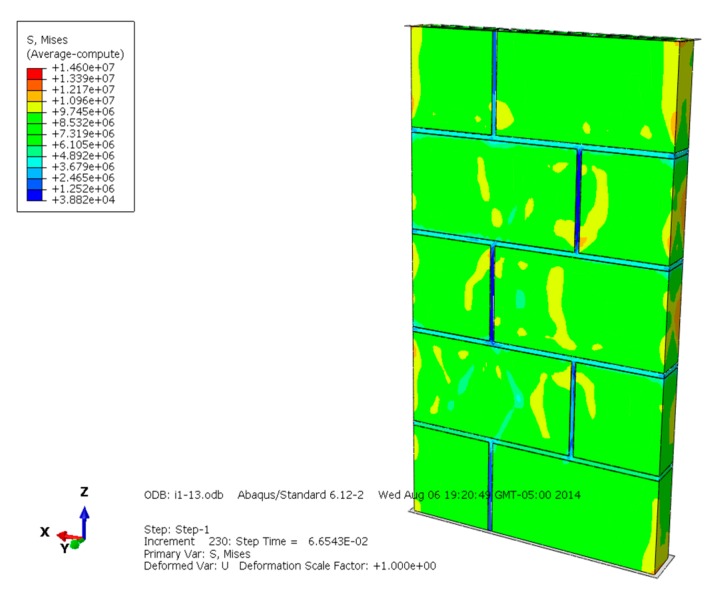
Mises equivalent stress of masonry constructed with M5 mortar.

**Figure 7:**
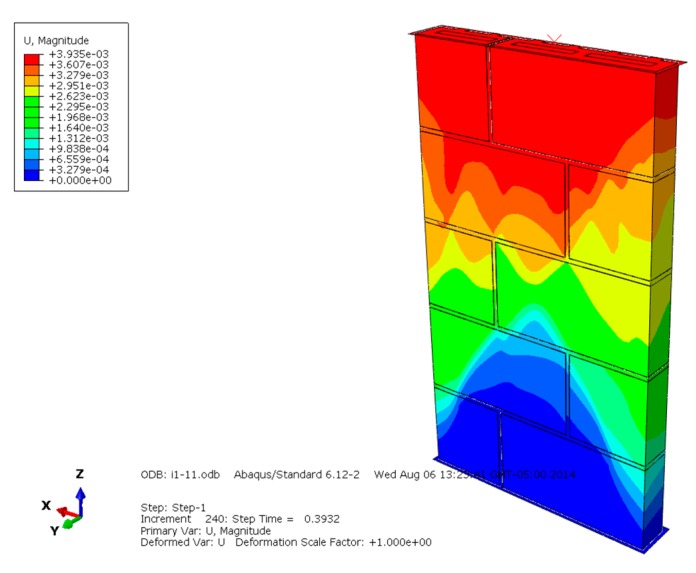
Displacement diagram of masonry constructed with M15 mortar.

**Figure 8:**
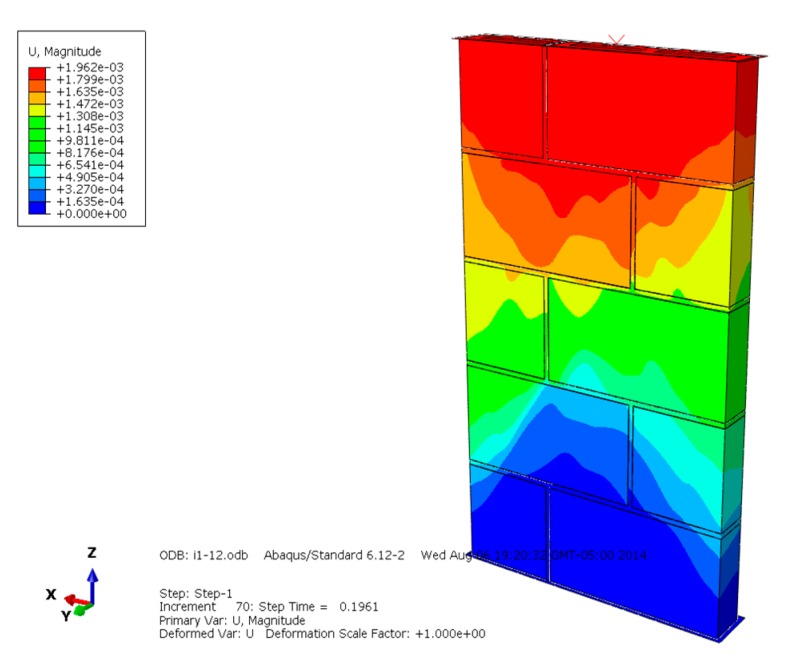
Displacement diagram of masonry constructed with M10 mortar.

**Figure 9:**
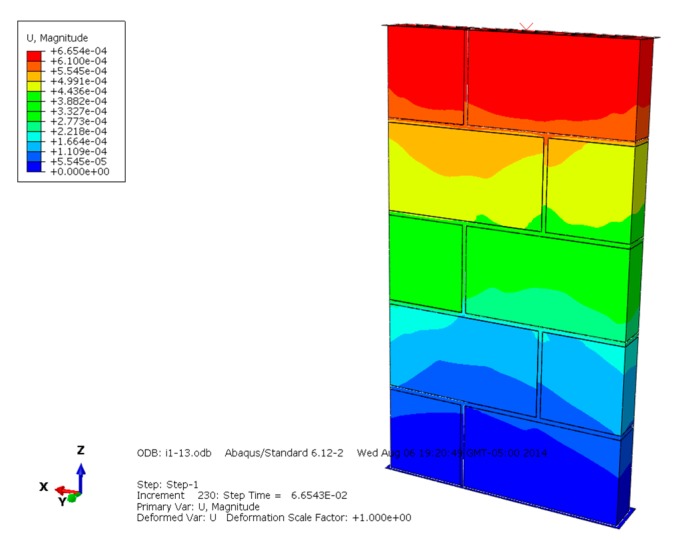
Displacement diagram of masonry constructed with M5 mortar.

**Figure 10:**
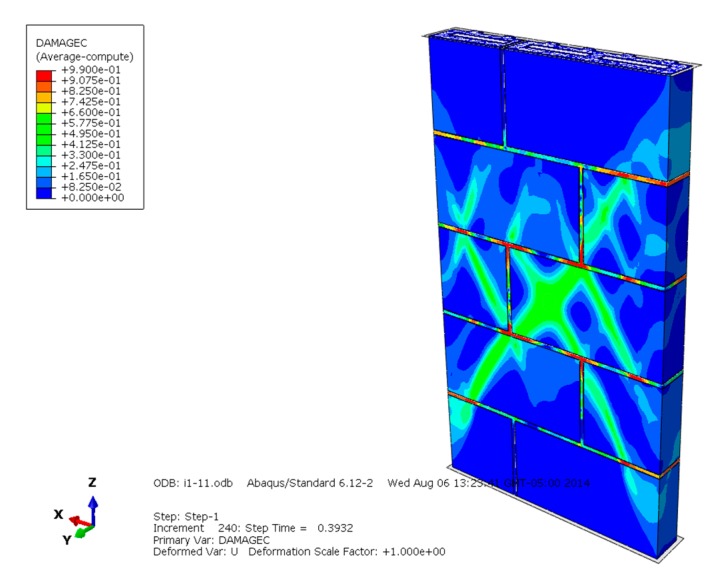
Compression damage diagram of masonry constructed with M15 mortar.

**Figure 11:**
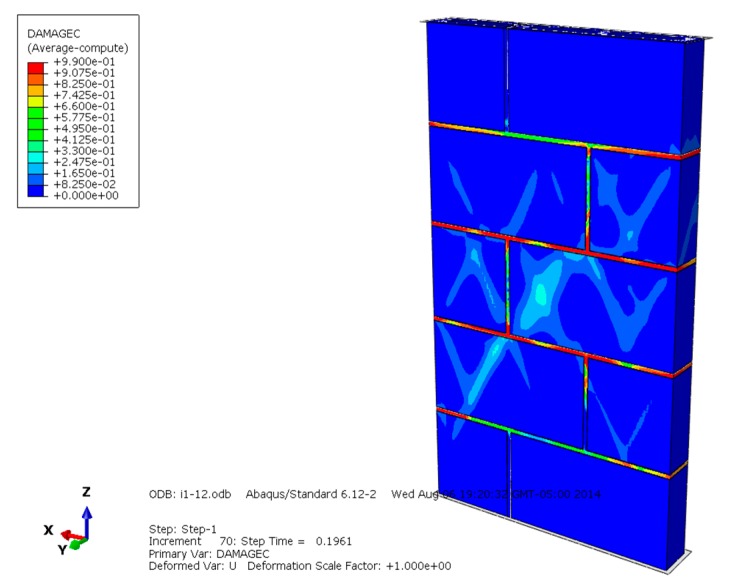
Compression damage diagram of masonry constructed with M10 mortar.

**Figure 12:**
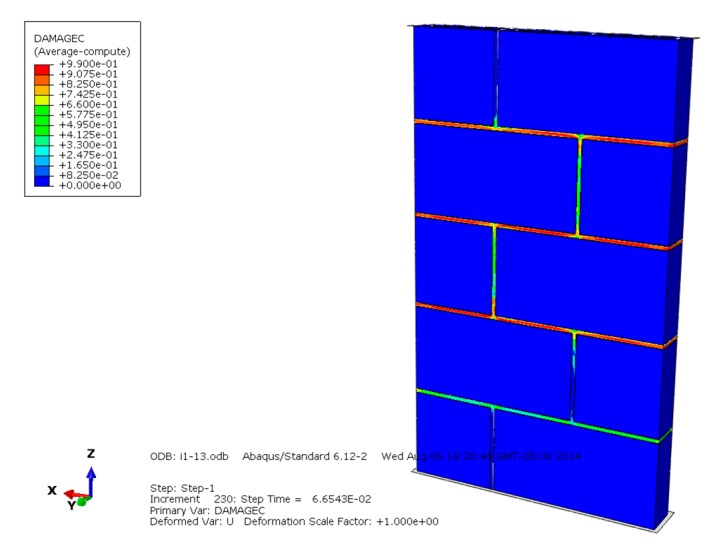
Compression damage diagram of masonry constructed with M5 mortar.

**Figure 13:**
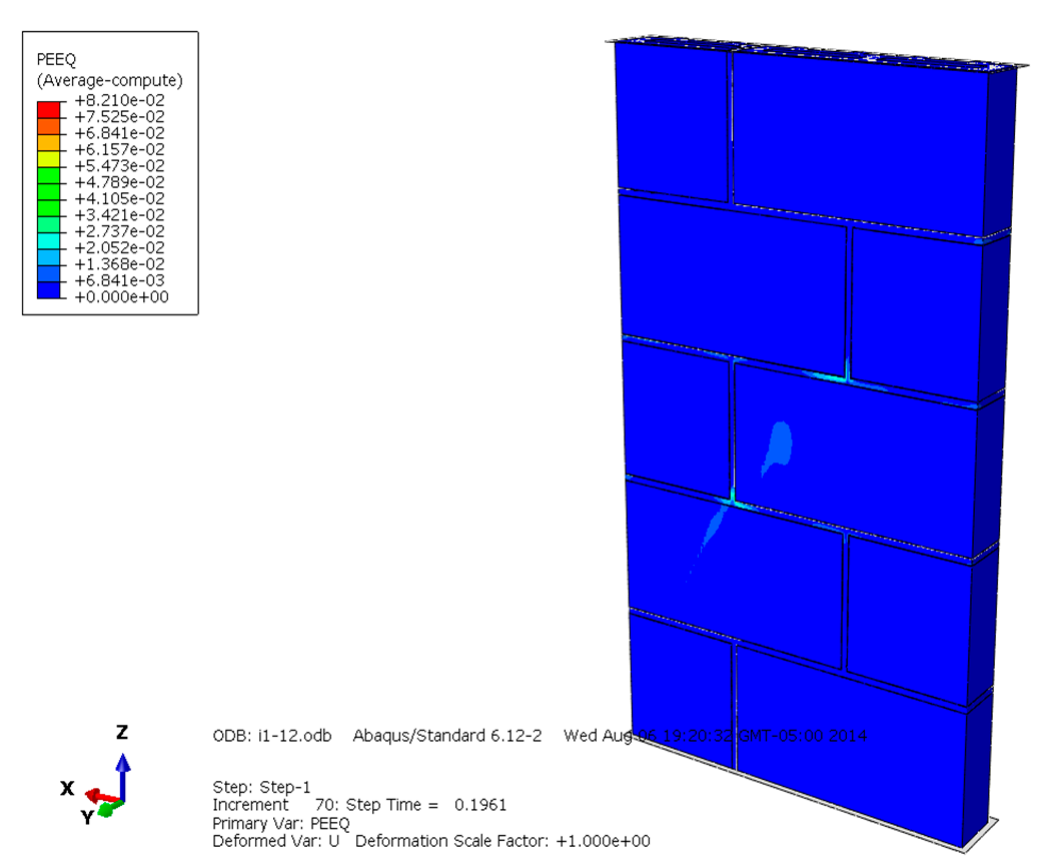
Plasticity diagram of masonry constructed with M15 mortar.

**Figure 14:**
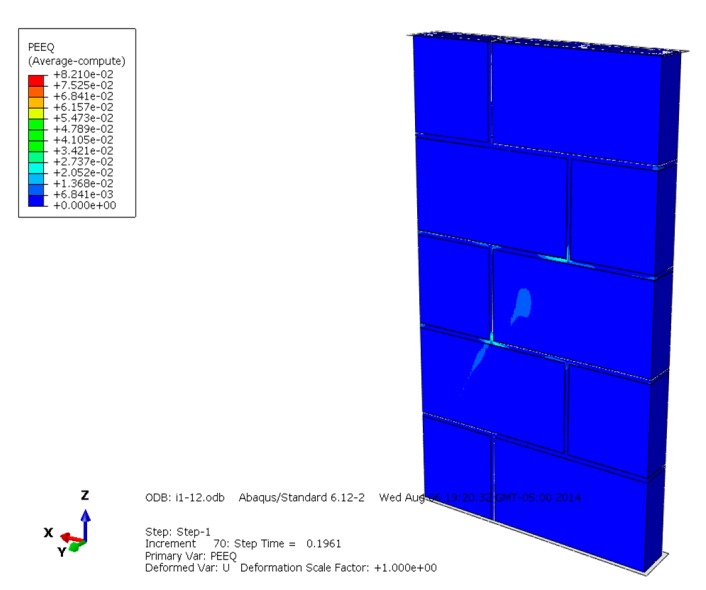
Plasticity diagram of masonry constructed with M10 mortar.

**Figure 15:**
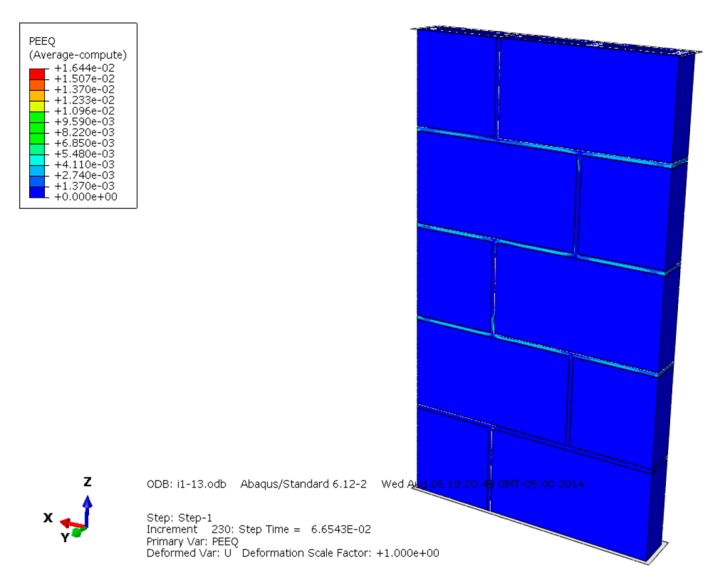
Plasticity diagram of masonry constructed with M5 mortar.

**Figure 16:**
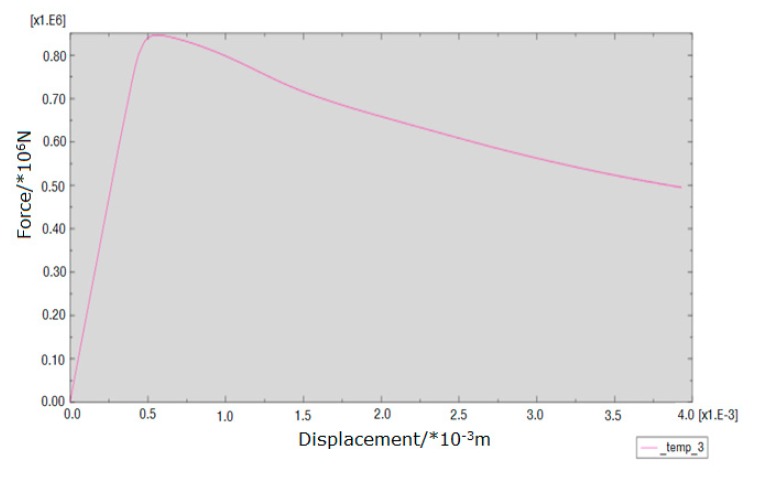
Load-displacement curve of masonry constructed with M15 mortar.

**Figure 17:**
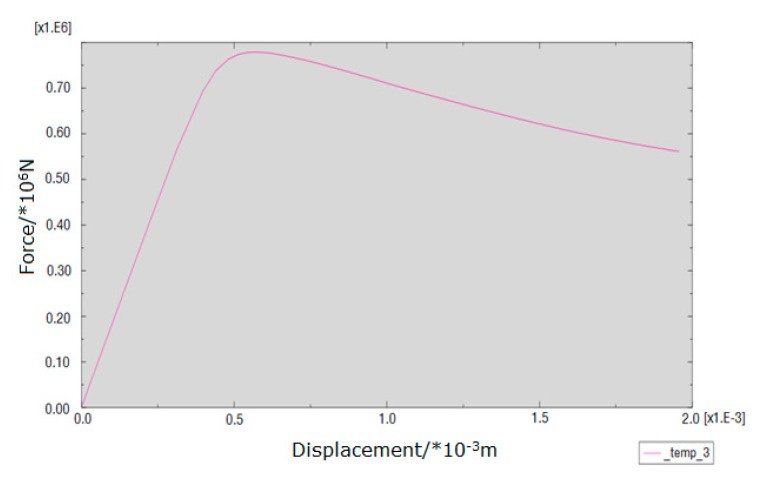
Load-displacement curve of masonry constructed with M10 mortar.

**Figure 18:**
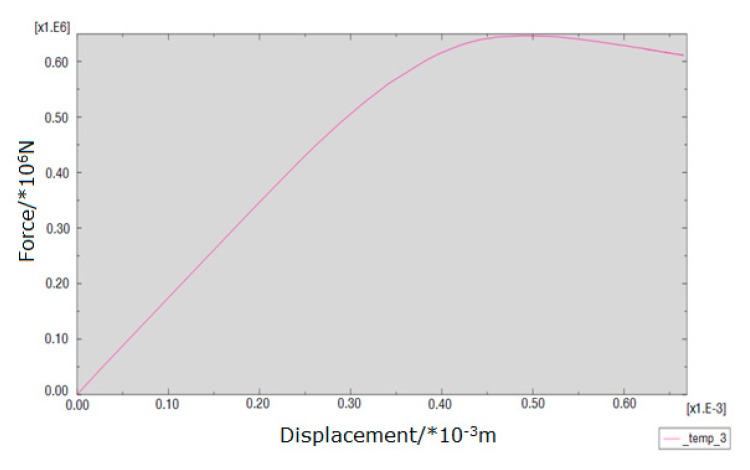
Load-displacement curve of masonry constructed with M5 mortar.

The Mises equivalent stress, displacement, compression failure, plastic strain, and loading-displacement curve of the masonry are selected and analyzed (Figure 4, Figure 5, Figure 6, Figure 7, Figure 8, Figure 9, Figure 10, Figure 11, Figure 12, Figure 13, Figure 14, Figure 15, Figure 16, Figure 17 and Figure 18). Figure 4, Figure 5 and Figure 6 are the Mises equivalent stress diagrams of masonry constructed by mortars of different compressive strengths. It can be seen from Figure 4, Figure 5 and Figure 6 that: (1) When the mortar compressive strength is higher than the recycled concrete and self-insulation block compressive strength, the greater Mises equivalent stress value only appears in the upper and lower parts of the masonry, indicating that the mortar can provide reliable bond strength and the overall compressive strength of the masonry is better. (2) When the compressive strength of the mortar and block is equal to the recycled concrete and self-insulation block compressive strength, the distribution of Mises equivalent stress is quite uniform; it shows that the mortar and block have good synergy when the masonry is compression damaged. (3) When the compressive strength of the mortar is lower than the recycled concrete and self-insulation block compressive strength, the larger Mises equivalent stress is centralized in the interface of the mortar and block, which indicates that the compression failure of the masonry is mainly caused by the interface cohesion loss. When mortar and block have similar compressive strength, their performance both can be fully developed, making the masonry have better load capacity and displacment capacity.

The displacement diagrams of masonry constructed by mortars of different compressive strengths are shown in Figure 7, Figure 8 and Figure 9 and show that the maximum displacement occurs at the upper loading position. The higher the compressive strength of the mortar, the larger the developing displacement region, and the greater the maximum displacement. The maximum displacement of different masonry indicates that higher mortar compressive strength is beneficial to improving the overall compressive and deformation capacity of masonry.

The compression failure diagrams of masonry constructed with mortars of different compressive strengths are shown in Figure 10, Figure 11 and Figure 12 and reveal that when the mortar compressive strength is higher than the recycled concrete and self-insulation block compressive strength, the maximum compressive damage is concentrated on the mortar, the larger damage value appears on the block, and the compression failure of the masonry is controlled by the lower compressive strength of the mortar and block. While the compressive strength of the mortar and block is equal to the block compressive strength, the maximum compressive failure value is concentrated in the mortar, and the larger damage value appears on the middle blocks of the masonry; the compression failure of the masonry is controlled by the lower compressive strength of the mortar and some middle blocks in the masonry. Under the condition that the mortar compressive strength is lower than the block compressive strength, the maximum compression failure is concentrated on the mortar, and the compression failure of the masonry is mainly controlled by the compressive strength of the mortar.

Figure 13, Figure 14 and Figure 15 are the plasticity diagrams of masonry constructed with mortars of different compressive strengths, and show that the plasticity zone distribution is in according with the Mises equivalent stress diagrams and compression failure distribution. When the masonry is damaged under compression loading, the plastic area of the masonry constructed with M15 mortar mainly appears on some blocks in the middle of the masonry, and the whole distribution shape is like an "X". The plastic zone of the masonry constructed with M10 mortar appears mainly in the mortar and some blocks in the middle part of the masonry, and the plastic zone of the masonry constructed with M5 mortar mainly appears on the mortar. Plastic zone distribution indicates that the compressive failure of masonry with mortars of different compressive strengths has different development rules, which is worth further discussion.

The loading force vs. displacement of the center point in the upper surface curve of different kinds of masonry is shown in Figure 16, Figure 17 and Figure 18. It is known that the ultimate compressive load corresponding displacement of the three types of masonry is around 0.5 mm. After reaching the ultimate load, the compressive load of the masonry gradually decreases with the emergence of cracks, and the displacement of the masonry increases gradually until the masonry is completely destroyed. The compressive strength of masonry constructed with M15, M10, and M5 mortar is 6.55, 6.03, and 5 MPa, respectively. This shows that mortar compressive strength affects the compressive strength of masonry. It should be noted that, in the engineering application, the actual mortar strength is usually higher than the standard strength used in the design, which will make the compressive strength of the masonry structure higher than the numerical simulation results. When the compressive strength of the mortar is low, the compressive strength of the masonry is controlled by the mortar compressive strength. It is beneficial for improving the compressive strength of masonry by improving the compressive strength of the mortar, but it has a limited effect when the compressive strength of the mortar is higher than the compressive strength of the block. For the 10.6 MPa compressive strength block, when the compressive strength of the mortar is increased from M10 to M15, the compressive strength of the mortar is increased by 50%, but the compressive strength of the masonry is increased by only 8.6%.

## 4. Experimental Verification

**Figure 19:**
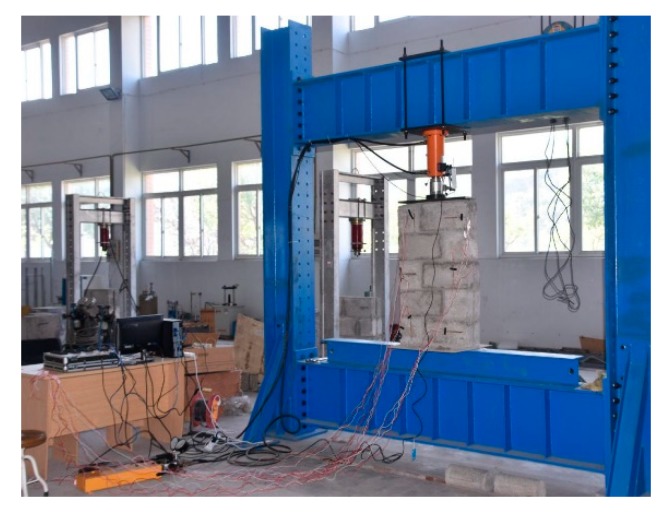
Picture of experimental test.

**Figure 20:**
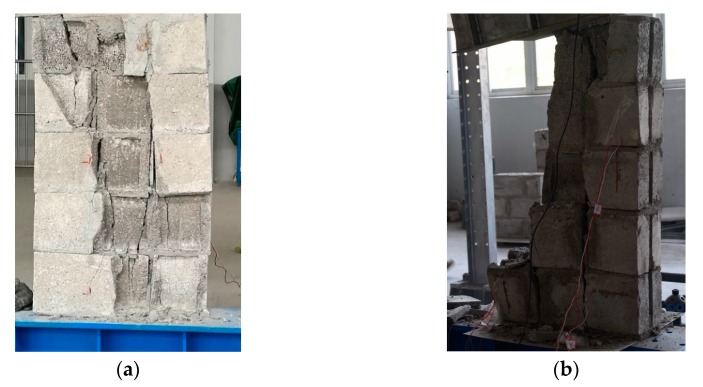
Damage to masonry constructed with M15 mortar: (**a**) Partial destruction and (**b**) Total destruction.

**Figure 21:**
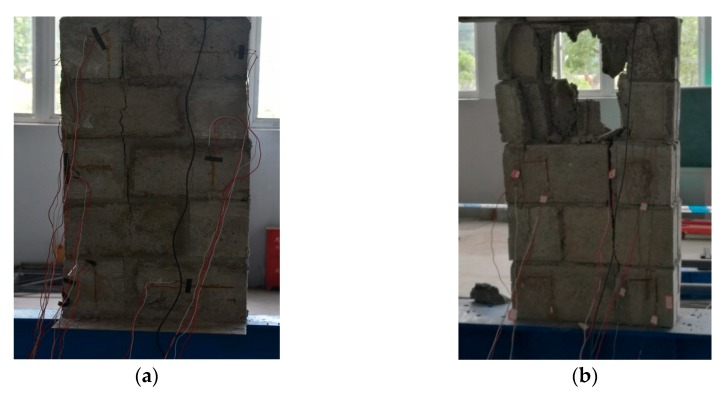
Damage to masonry constructed with M10 mortar: (**a**) Crack development and (**b**) total destruction.

**Figure 22:**
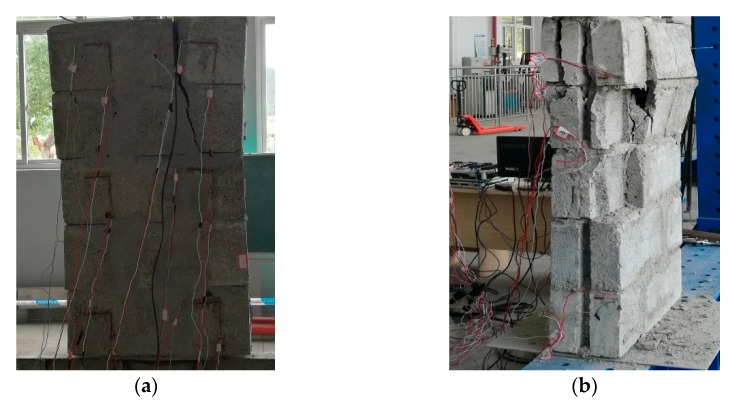
Damage to masonry constructed with M5 mortar: (**a**) Crack development and (**b**) total destruction.

**Figure 23:**
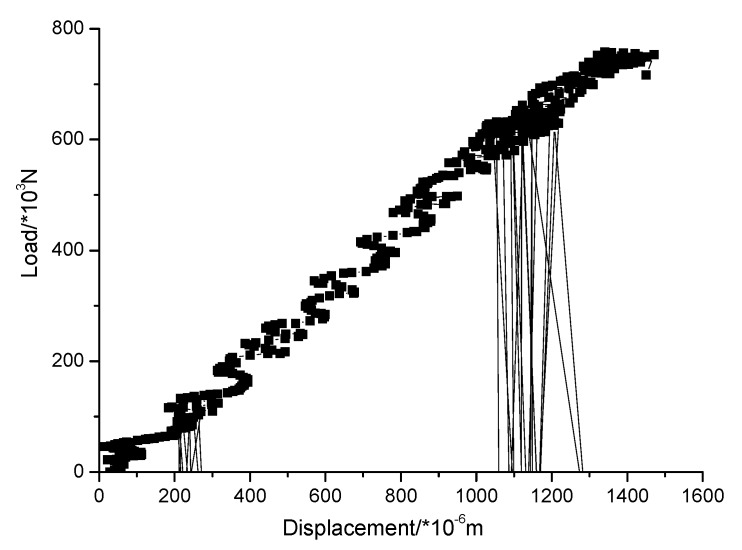
Load–displacement curve of masonry constructed with M15 mortar.

**Figure 24:**
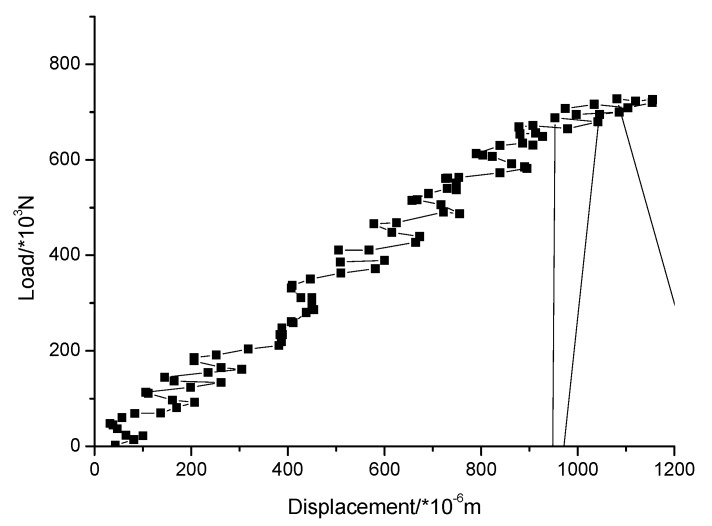
Load–displacement curve of masonry constructed with M10 mortar.

**Figure 25:**
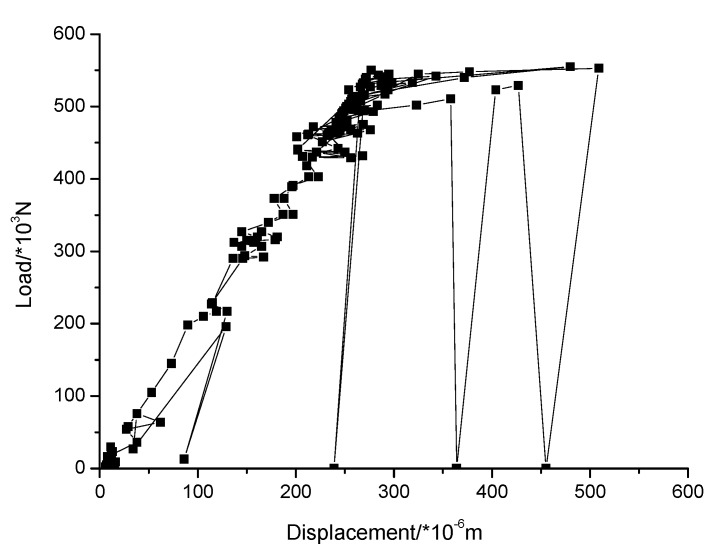
Load–displacement curve of masonry constructed with M5 mortar.

In order to verify the rationality of the ABAQUS numerical simulation results, the compressive properties of masonry constructed with 10.6 MPa compressive strength recycled concrete and self-insulation blocks and M15, M10, and M5 compressive strength mortar are experimentally tested. The measured compressive strength of M15, M10, and M5 mortar are 15.1, 8.7, and 4.6 MPa, respectively. The experimental device consisted of a rigid frame counterforce device, a pressurizing device, and a measuring system device. For details of the experimental device, see Figure 19. The displacement of the upper surface of the masonry was measured. The damage mechanisms of masonry constructed with M15, M10, and M5 mortar are shown in Figure 20, Figure 21 and Figure 22, and the load–displacement curves of different types of masonry are shown in Figure 23, Figure 24 and Figure 25.

One can see from Figure 20, Figure 21 and Figure 22 that when the mortar has a higher compressive strength than the blocks, the blocks crack after the masonry is loaded to some extent. With the increase of load and the development of block cracks, the masonry will suddenly collapse and be destroyed when the ultimate load is reached. When the compressive strength of the mortar and the blocks is similar, the masonry failure is composed of mortar failure and block fracture failure, and the masonry damage can be characterized as a splitting fracture. When the mortar has a lower compressive strength than the blocks, the failure of the masonry is mainly caused by the insufficient bonding strength of the mortar; the masonry damage mode is that the block in the middle of the masonry is falling, and the upper block is crushed.

Figure 23, Figure 24 and Figure 25 show that the damage to different masonry is brittle failure. It is worth pointing out that the lines connecting some points of the graph with the displacement axis denote crack development in the wall. During the compression process of the M15 mortar, the block first cracks when the deformation is 0.5 mm; because of the higher mortar compressive strength, the masonry still maintains a good integrity and can continue to bear the load. As the load is further increased, block cracks develop, and when the masonry is loaded to about 755 KN, a large number of cracks appear and develop, then the masonry suddenly collapses. When the compressive strength of the mortar is slightly lower than the compressive strength of the blocks, the damage to the masonry is mainly controlled by the bond strength of the mortar. For masonry constructed with M5 mortar, when the load increases to 693 KN, the masonry begins to break through cracks; the length and width of the cracks develop continuously with the increase of the load, which eventually leads to damage to the masonry. It is necessary to mention that the tested masonry was constructed by the research team members, and the masonry quality is general and may affect masonry compression performance to a certain extent, which could lead to slight difference in the experimental and numerical simulation results for the masonry that is constructed with M10 mortar, but the overall trend of them all is consistent.

## 5. Discussion

For improving building energy saving level and achieving the utilization of waste concrete and zinc and lead tailings, a self-insulation block with a three-row-hole arrangement was developed using recycled concrete broken aggregate and activated zinc and lead tailings. The rational selection of the block and mortar constitutive model and corresponding parameters in the ABAQUS numerical simulation were theoretically analyzed, and different masonry was constructed using 10.6 MPa compressive strength blocks and M15, M10, and M5 mortar. The compression performance of the masonry was numerically analyzed by ABAQUS and experimentally tested. Through the analysis of numerical simulation and experimental results, the following two points need to be further discussed.

(1) The stress-strain curve of masonry constructed with mortars of different compressive strengths. The experimental study of Corradi et al. showed that the relationship of compressive vertical stress and strain is an approximate linear [16], but the experimental compressive vertical stress used was only 0.33 MPa and the corresponding strain values were relatively small, which is in good agreement with the experimental results in this paper. Kurdo F. Abdulla et al. simulated masonry wall behavior using a simplified micro-model approach [17]; the experimental and numerical simulation results showed that the compressive strength of masonry is similar to the results of the numerical simulation in this paper, but their stress-strain curve descent was faster after stress reached the maximum. The research of Sarangapani et al. indicated that an increase in bond strength, while keeping the mortar strength constant, lead to an increase in the compressive strength of masonry [18].

It is necessary to mention that, in this experiment, the compressive vertical stress was loaded in a few minutes, and the loading was rigid because the servo-loading system was not used. In this case, the plastic strain of masonry constructed by mortars of different compressive strengths could not be fully developed. The law of strain development and the nonlinear plasticity assumed by the numerical simulation are a little different, which caused the load-displacement curves of the numerical simulation and the experimental tests to be different. For the load-displacement curve of the numerical simulation, the strain of the masonry constructed with mortars of each compressive strength is about 0.5 mm when the load reaches the maximum, but, for the measured load displacement curve, due to the appearance and development of the local cracks, the strain value under the ultimate load of the masonry constructed by mortars of higher compressive strengths increases. For instance, for masonry constructed with M10 and M15 mortar, the strain values corresponding to the ultimate load were 1 mm and 1.2 mm, respectively.

It can be seen that the numerical simulation results of the ultimate load are consistent with the experimental results, but the numerical simulation results of the deformation value of masonry structures under the ultimate load are quite different from the experimental results. This is mainly because of the selection of the numerical simulation parameters, and the constitutive model of this new type of masonry needs to be further discussed. The damage diagrams are not consistent because the compression cracks are generally parallel to the load direction, which might be due to the deficiency of the masonry walls because of the insufficient technological level of the laboratory staff. More experimental data need to be accumulated for obtaining reasonable numerical simulation results by adopting parameters that are consistent with the experimental results, which is also the direction of further research.

(2) The compressive strength of masonry constructed with different mortars. According to the National Standard of the People’s Republic of China, Appendix B of the Code for the design of masonry structures [19], the average axial compression strength value of different masonry can be calculated by Equation (5).

(5) $$f_{m}=k_{1}\;f_{1}^{\alpha }\;(1+0.07f_{2})\;k_{2}$$

Here, $f_{1}$ is the block’s compressive strength grade value, MPa, $f_{2}$ is the average compressive strength value of the mortar, and $f_{m}$ is the average axial compressive strength of the concrete block masonry. If $f_{2}$ > 10 MPa, $f_{m}$ should be multiplied by the coefficient 1.1–0.01$f_{2}$. For the concrete block, the coefficients $k_{1}$, α, and $k_{2}$ are 0.46, 0.9, and 1.0, respectively.

Calculated by Equation (5), the average compressive strength of masonry constructed with M15, M10, and M5 compressive strength mortar should be 7.39, 6.09, and 5.0 MPa, respectively. The calculated value is slightly higher than the experimentally measured value for masonry that is constructed with M15 mortar, which may be due to the fact that the masonry quality is not up to the average level or brick-mortar bond strength difference [18]. The details need further experimental testing and discussion.

## 6. Conclusions

By using recycled concrete aggregate and activated zinc and lead tailing powder, the authors developed a new type of three-row-hole block, which had a better energy saving effect. Based on previous research, this paper studied the compressive strength and displacement of masonry constructed with this kind of block and mortars of different compressive strengths through ABAQUS numerical simulation analysis, and compared them with the experimental results. The following conclusions could be drawn from the above mentioned research:

(1) When the mortar compressive strength is higher than the compressive strength of the blocks, because it can provide reliable bond strength, masonry compression is determined by the block compressive strength, the masonry can bear larger displacement, the overall compressive performance is better, and the higher mortar compressive strength is beneficial to improve the overall compressive and deformability of the masonry.
(2) When the compressive strength of mortar and block is equal, the masonry can bear a certain deformation and the mortar and block have good synergy. The compression failure of the masonry is controlled by the lower compressive strength of the local block in the middle of the masonry and by the mortar.
(3) When the mortar compressive strength is lower than the compressive strength of the blocks, the compression failure of the masonry is mainly caused by the interface bond loss, the deformation performance of the masonry is poor, and the compression failure of the masonry is mainly controlled by the mortar compressive strength.
(4) Under the reasonable constitutive model and parameter selection, the ABAQUS numerical simulation can obtain satisfactory results. The masonry quality has a certain influence on the measured results. The effect of the masonry quality and the loading mode on the compressive strength of the masonry should be considered when the results of the numerical simulation are applied to the engineering application.
